# Longitudinal associations between parental trauma-related symptoms and adolescent emotion regulation: a three-wave dyadic analysis

**DOI:** 10.3389/fpsyg.2026.1882113

**Published:** 2026-10-07

**Authors:** Shooroq Maberah, Mohammed Abu Al-Rub

**Affiliations:** 1 Department of Psychological and Family Counseling, College of Educational Sciences, Jadara University, Irbid, Jordan; 2 Department of Special Education, Faculty of Educational Sciences, Jadara University, Irbid, Jordan

**Keywords:** actor-partner interdependence model, emotion regulation, intergenerational trauma, longitudinal dyadic design, refugee mental health

## Abstract

**Introduction:**

Intergenerational trauma research has often treated transmission as unidirectional and static, overlooking dyadic and reciprocal processes. This study applied a three-wave longitudinal Actor–Partner Interdependence Model (APIM) to examine bidirectional associations between parental trauma-related symptoms and adolescent emotion regulation (ER) difficulties.

**Methods:**

A total of 247 parent–adolescent dyads from Syrian refugee and Jordanian host-community families in Jordan completed three waves over six months. Parents reported lifetime trauma at T1; both parents and adolescents reported ER difficulties at T1, T2, and T3. Distinguishable-dyad APIMs estimated autoregressive, actor, and partner effects with FIML and covariates.

**Results:**

Parental trauma at T1 significantly predicted offspring ER difficulties at T2 (β = 0.23), mediated longitudinally by parent ER at T2. Offspring ER at T1 also predicted parent ER at T2 (β = 0.12), but this child-driven effect did not persist to T3. Refugee status did not moderate the transmission paths.

**Discussion:**

Intergenerational trauma appears to operate as a dynamically co-constructed dyadic process. Findings support process-focused, dyadic interventions rather than treating adolescents in isolation.

## 1 Introduction

The understanding of psychological trauma has shifted over the past decade: from a time-bound event to a potential source of intergenerational vulnerability. A core insight of contemporary research is that parental trauma-related symptoms may persist and influence caregiving and relational processes (Yehuda et al., 2016; Yehuda and Lehrner, 2018). Longitudinal studies of Holocaust survivors, refugees, and maltreated samples consistently show elevated psychopathology in offspring who experienced no direct trauma (Dalgaard et al., 2020; Dashorst et al., 2019). Yet most existing studies treat transmission as unidirectional and static, ignoring the recursive dynamics through which parental experiences and offspring behaviors shape each other over time (Klahr and Burt, 2014).

However, it is essential to distinguish between trauma exposure itself, the PTSD symptoms that may follow, and the emotion regulation difficulties that may arise in offspring. These are conceptually distinct constructs that should not be used interchangeably (Isobel et al., 2019). The present study does not claim to measure trauma transmission directly; rather, we examine prospective associations between parental trauma-related symptom severity and adolescent emotion regulation difficulties, while acknowledging that multiple unmeasured mechanisms may account for these associations.

Among the proposed mechanisms, offspring emotion regulation (ER) is the most empirically supported pathway. Parental unresolved trauma predicts reduced emotional availability, increased irritability, and inconsistent responses to child distress—each undermining the child’s acquisition of flexible ER strategies (Clark et al., 2021; Morris et al., 2024). Furthermore, a critical synthesis by Isobel et al. (2019) emphasizes that parental trauma history disrupts the relational and regulatory environment, leading to impaired emotion regulation capabilities in offspring when parents are unable to provide secure scaffolding. However, nearly all included studies used single-informant questionnaires at one point, treating ER as a static trait rather than a dyadically co-constructed process. Moreover, the child’s influence on the parent—whether child dysregulation worsens parental symptoms, creating a feedback loop—remains understudied (Reitz et al., 2006). As Lobo and Lunkenheimer (2020) note, ER develops within reciprocal caregiver-child transactions; ignoring bidirectionality produces biased estimates of transmission.

The intergenerational trauma literature suffers from two methodological limitations. First, measurement bias: most studies rely on offspring retrospective reports or single-informant self-reports (Isobel et al., 2019; Hardt and Rutter, 2004). This conflates perceived memory with observed interaction, introduces shared method variance, and cannot separate parent-driven from child-driven effects. Second, temporal myopia: cross-sectional designs cannot establish directionality, and two-wave designs measure change but not the mutual regulatory dynamics (reciprocal influence, feedback loops, time-lagged partner effects) that unfold over months (Little, 2022). As Hamaker et al. (2015) showed, detecting bidirectional influence requires at least three waves to separate cross-lagged paths from autoregressive stability and within-wave residual covariance. High-impact journals increasingly reject single-wave or two-wave dyadic studies on intergenerational transmission because they cannot rule out reverse causation or spurious associations (Kenny et al., 2006). A study aiming to capture mutual dynamics must therefore: (a) collect parallel data from parents and offspring; (b) use three or more waves (e.g., September baseline, December follow-up, March second follow-up); and (c) apply a model that partitions variance into actor and partner effects while modeling bidirectional lags.

The present study introduces this innovation: the Actor-Partner Interdependence Model (APIM) applied to three-wave longitudinal dyadic data. Originally developed by Kenny and Ledermann (2010), APIM has been extended to parent–child dyads and explicitly models interdependence by estimating actor effects (the impact of one’s own prior trauma or ER on their subsequent outcomes) and partner effects (cross-person, cross-time influence) (Cook and Kenny, 2005). Unlike conventional regression that assumes independence, APIM provides unbiased estimates of reciprocal influence while controlling for autoregressive paths. Methodological advancements in APIM (Cook and Kenny, 2005) show that longitudinal designs allow testing not only whether a parent’s trauma predicts the child’s ER six months later (partner effect), but also whether the child’s ER reciprocally predicts the parent’s subsequent adjustment—capturing the true mutual dynamics of transmission. To date, no study of intergenerational trauma and offspring ER has used three-wave APIM with both generations assessed simultaneously. This gap is striking given that theoretical models have long emphasized bidirectionality (Reitz et al., 2006; Bowers and Yehuda, 2016).

Accordingly, this study pursues three objectives: (1) to test the bidirectional pathways between parental unresolved traumatic experiences and offspring emotion regulation using three-wave longitudinal dyadic APIM; (2) to quantify actor effects (intrapersonal continuity within parents and within children) and partner effects (cross-generational influence) while controlling for autoregressive stability; and (3) to examine whether partner effects are asymmetric (i.e., parent→child effects differ in magnitude from child→parent effects). By moving beyond static, unidirectional designs and adopting a temporally dense dyadic approach, this study resolves a key impasse in the intergenerational trauma literature and provides empirically grounded targets for family-based prevention.

## 2 Methodology

### 2.1 Study design and participants

This study employed a three-wave longitudinal dyadic design targeting parent-offspring pairs (dyads). Eligibility criteria were: (a) the parent reported exposure to at least one traumatic event (Criterion A for PTSD per DSM-5-TR) (American Psychiatric Association, 2022); (b) the offspring was aged 12–17 years (adolescence, a critical period for emotion regulation consolidation); (c) both parent and offspring resided in the same household with regular contact (≥4 days/week); (d) no current psychotic disorder or active substance dependence in either dyad member that would preclude valid self-report.

Dyads were recruited between June and August 2025 from community mental health centers, refugee support programs (including Syrian refugee families registered with UNHCR Jordan), and general population advertisements in the northern region of Jordan (Irbid Governorate and surrounding areas). This geographic focus provided access to a population exposed to high rates of collective trauma (war, displacement) and ongoing socioeconomic stressors, while remaining logistically feasible for three-wave follow-up. The cultural context characterized by extended family interdependence and specific emotion-display rules offers a valuable counterpoint to the predominantly WEIRD samples (Western, Educated, Industrialized, Rich, Democratic) in the intergenerational trauma literature (Henrich et al., 2010). Written informed consent was obtained from parents and assent from offspring in Arabic.

The final sample comprised *N* = 247 dyads (494 individuals) after excluding three dyads that withdrew before the first assessment. This sample size was based on a power analysis for APIM with three waves: detecting a small-to-moderate partner effect (*β* ≥ 0.15) with 80% power at *α* = 0.05 requires approximately 220 dyads when assuming an intraclass correlation *ρ* = 0.30 within dyads (Ackerman et al., 2017). We initially recruited 250 dyads to account for 12% attrition; the actual retention rate was 94.4% (236 dyads completed all three waves).

### 2.2 Temporal protocol (three waves)

Data collection occurred at three time points with a fixed interval of 3 months between waves, chosen to capture developmental changes in emotion regulation while remaining sensitive to potential therapeutic or environmental fluctuations, and because 3 months provides sufficient time for cumulative daily interactions to manifest trait-like score changes without extreme maturation effects.

#### 2.2.1 Wave 1 (T1; September 2025, baseline)

Parents completed the PTSD Checklist for DSM-5 (PCL-5, lifetime version) and the Difficulties in Emotion Regulation Scale -- Short Form (DERS-SF). Offspring completed the DERS-SF and provided baseline data on potential covariates (age, gender, time since the parent’s most recent trauma). Both dyad members completed the assessments independently in separate rooms to minimize cross-influence.

#### 2.2.2 Wave 2 (T2; December 2025, first follow-up)

Both parents and offspring re-completed the DERS-SF. Parents also reported any intervening stressful life events using the Life Events Checklist to identify potential confounds.

#### 2.2.3 Wave 3 (T3; March 2026, second follow-up)

Final administration of the DERS-SF to both dyad members. Additionally, offspring completed the Strengths and Difficulties Questionnaire (SDQ) to characterize clinical significance.

Each wave lasted approximately 45 min. Missing data were handled using Full Information Maximum Likelihood (FIML) in AMOS, which yields unbiased parameter estimates under the missing-at-random assumption (Little, 2022). Attrition analysis showed no significant differences between completers (*n* = 236) and non-completers (*n* = 11) on any baseline variable (all *p* > 0.10).

Parental trauma-related symptoms were assessed only at T1 (baseline) using the lifetime version of the PCL-5, which assesses symptoms over the past month. While this provides a measure of current trauma-related symptom severity, it does not capture changes in trauma-related symptoms over time. Consequently, the temporal ordering between parental trauma-related symptoms and subsequent changes in ER difficulties cannot be fully established. The longitudinal design primarily captures the temporal relationship between baseline parental symptoms and changes in ER over the subsequent six months, but we cannot determine whether changes in parental symptoms preceded changes in ER at specific time points. We acknowledge this limitation in the Discussion.

### 2.3 Measures

All instruments were administered in Arabic, using validated translations. Internal consistency (Cronbach’s α and McDonald’s ω) was computed for each scale in the current sample.

#### 2.3.1 Parental traumatic experiences (T1 only)

The PTSD Checklist for DSM-5 (PCL-5) a 20-item self-report measure assessing exposure to traumatic events (Criterion A) and the severity of PTSD symptoms in the past month. The lifetime version was used to capture the presence and severity of unresolved trauma. The PCL-5 has excellent internal consistency (*α* > 0.94) and test–retest reliability (*r* = 0.82) (Blevins et al., 2015). The Arabic version has shown acceptable psychometric properties in refugee and community samples (Ibrahim et al., 2018). However, we acknowledge that the PCL-5 has not been specifically validated in the Syrian refugee or Jordanian host-community populations included in this study. In the present sample, *α* = 0.93. We also recorded the number of distinct trauma types (e.g., physical assault, accident, war-related trauma).

#### 2.3.2 Emotion regulation—parent and offspring (assessed at T1, T2, T3)

The Difficulties in Emotion Regulation Scale Short Form (DERS-SF) an 18-item scale measuring six dimensions of emotion regulation difficulties: non-acceptance of emotional responses, difficulties engaging in goal-directed behavior, impulse control difficulties, lack of emotional awareness, limited access to emotion regulation strategies, and lack of emotional clarity. The DERS-SF has strong psychometric properties (*α* = 0.88–0.93) and is suitable for adolescents (≥12 years) and adults (Hallion et al., 2018). An Arabic version is validated (El-Ghossain et al., 2020). While the Arabic version of the DERS-SF has shown acceptable psychometric properties in general Arabic-speaking samples, it has not been specifically validated with Jordanian adolescents or parents. The use of instruments validated in broadly similar but not identical populations may introduce measurement bias, particularly for culturally specific expressions of distress or emotion regulation difficulties. In the current sample, α ranged from 0.86 to 0.91 across waves for parents and offspring. For offspring, the total ER difficulty score was used as the primary outcome.

#### 2.3.3 Covariates (assessed at T1)

Offspring age and gender, parent gender, number of years since the parent’s most recent trauma, family socioeconomic status (monthly household income and parent education), offspring mental health treatment in the past six months (yes/no), and refugee status (Syrian refugee vs. Jordanian citizen). We acknowledge that our covariate set was limited to demographic factors and refugee status, and did not include measures of parental depression, anxiety, current stress, family conflict, parenting behavior, or emotional availability. These unmeasured constructs represent important potential confounders that could account for some or all of the observed associations. We address this limitation in the Discussion.

### 2.4 Dyadic structure and model specification

Dyads were structurally treated as distinguishable based on generational roles (parent vs. offspring). This decision reflected theoretical and empirical reality: parents and adolescents systematically differ in mean levels, variances, and temporal stability of emotion regulation difficulties (Little, 2022; Cook and Kenny, 2005). In the Actor-Partner Interdependence Model (APIM) implemented in IBM AMOS 29, means, variances, and all actor/partner paths were freely estimated for each member rather than constrained to equality. Treating these dyads as indistinguishable would have imposed invalid equality constraints (e.g., forcing parent ER variance = offspring ER variance) and biased standard errors. Thus, the distinguishable specification was used throughout.

The analytical framework was cross-lagged structural equation modeling (SEM) within APIM extended to three time points, estimated using maximum likelihood with FIML. The path diagram included the following components:

1 Autoregressive paths (stability over time):

- Parent: Parent ER(T1) → Parent ER(T2); Parent ER(T2) → Parent ER(T3)
- Offspring: Offspring ER(T1) → Offspring ER(T2); Offspring ER(T2) → Offspring ER(T3)

2 Actor effects (cross-lagged, within person):

- Parent trauma (T1) → Parent ER(T2)
- Parent ER(T1) → Parent ER(T3) (indirect via T2 captured in autoregressive cascade)
- Offspring ER(T1) → Offspring ER(T2) and ER(T2) → ER(T3) were accounted for in autoregressive paths; the specific actor effect of trauma on later ER was estimated only for parents because offspring trauma was not measured at T1.

3 Partner effects (cross-lagged, cross-person -- the core intergenerational transmission):

- Parent trauma (T1) → Offspring ER(T2)
- Parent ER(T1) → Offspring ER(T2)
- Parent ER(T2) → Offspring ER(T3)
- *Bidirectional partner effect*: Offspring ER(T1) → Parent ER(T2) (testing child-driven regulation of parent)
- Exploratory long-lag effect: Parent trauma (T1) → Offspring ER(T3)

Crucially, we acknowledge that the parent-to-offspring and offspring-to-parent pathways are not symmetrical estimates of the same dyadic process. The parent-to-offspring pathway examines whether parental trauma-related symptoms predict offspring ER difficulties, whereas the offspring-to-parent pathway examines whether offspring ER difficulties predict parental ER difficulties. These involve different predictor variables and therefore cannot be interpreted as equivalent transmission effects. We characterize this as an examination of bidirectional associations in emotion regulation processes, not bidirectional trauma transmission.

4 Within-wave covariances: Residuals of parent ER and offspring ER were correlated at T1, T2, and T3 to account for shared environmental influences or measurement occasion effects.

All paths were estimated separately for parents and offspring, consistent with the distinguishable dyad specification. The model was identified because each wave provided sufficient covariance information.

### 2.5 Hypotheses

The following hypotheses were formulated and tested:

- H1 (Autoregressive stability): Each dyad member’s emotion regulation difficulties would show moderate to high stability from T1 to T2 and T2 to T3 (standardized *β* ≥ 0.40, *p* < 0.01).
- H2 (Actor effect): Parental trauma-related symptom severity at T1 would positively predict parents’ own ER difficulties at T2, controlling parent ER at T1 (*β* > 0, *p* < 0.05).
- H3 (Partner effect - parent-to-offspring): Parental trauma-related symptoms at T1 would positively predict offspring ER difficulties at T2, after controlling for offspring ER at T1 and demographic covariates (*β* > 0, *p* < 0.01). This provides evidence of a prospective association between parental trauma-related symptoms and offspring regulatory outcomes.
- H4 (Bidirectional association - offspring-to-parent): Offspring ER difficulties at T1 would positively predict parent ER difficulties at T2, indicating mutual dynamics in emotion regulation (*β* > 0, *p* < 0.05). This tests whether adolescent dysregulation is associated with changes in parental regulation, but it does not constitute evidence of upward trauma transmission.
- H5 (Longitudinal cascade): Parent ER difficulties at T2 would mediate the relationship between parent trauma at T1 and offspring ER difficulties at T3; similarly, offspring ER at T2 would mediate the relationship between offspring ER at T1 and parent ER at T3.

### 2.6 Data analysis plan

Preliminary analyses were conducted using SPSS 29. Missing data patterns were examined with Little’s MCAR test (*χ^2^* = 142.3, *df* = 135, *p* = 0.32), indicating that data were missing completely at random. Normality was assessed (skewness < |2|, kurtosis < |7| for all variables). Descriptive statistics (means, standard deviations, correlations) were computed for all study variables at each wave. Attrition vs. retained dyads were compared on baseline variables using independent t-tests and *χ^2^* tests; no significant differences emerged.

#### 2.6.1 Longitudinal measurement invariance

To ensure that the DERS-SF measured the same construct consistently across time, we tested longitudinal measurement invariance separately for parents and adolescents across the three waves (T1, T2, T3). Following established guidelines (Putnick and Bornstein, 2016), we tested three levels of invariance using confirmatory factor analysis in AMOS 29:

- Configural invariance: the same factor structure (one-factor model with 18 items) was specified freely across waves.
- Metric invariance: factor loadings were constrained to equality across waves.
- Scalar invariance: item intercepts were additionally constrained to equality across waves.

### 2.7 Measurement invariance

Model fit was evaluated using CFI ≥ 0.90, RMSEA ≤ 0.08, and SRMR ≤ 0.08. Invariance was supported if ΔCFI ≤ 0.01 and ΔRMSEA ≤ 0.015 between nested models (Chen, 2007).

For parents, the configural model showed acceptable fit: *χ^2^* (1350) = 2145.6, CFI = 0.92, RMSEA = 0.058, SRM*R* = 0.051. The metric model (loadings constrained) showed ΔCFI = 0.004, ΔRMSEA = 0.002, supporting metric invariance. The scalar model (intercepts constrained) showed ΔCFI = 0.006, ΔRMSEA = 0.003, supporting scalar invariance.

For adolescents, similar results were obtained: configural model *χ^2^* (1350) = 2201.3, CFI = 0.91, RMSEA = 0.061, SRM*R* = 0.053; metric invariance: ΔCFI = 0.005, ΔRMSEA = 0.003; scalar invariance: ΔCFI = 0.007, ΔRMSEA = 0.004.

These results support full scalar invariance for both parents and adolescents, indicating that the DERS-SF measured ER difficulties consistently across time. Thus, observed changes in mean levels and cross-lagged associations can be interpreted as reflecting genuine changes in the underlying construct rather than measurement artifacts.

Cross-generational measurement invariance was also examined to determine whether parents and adolescents interpreted DERS-SF items similarly. Comparing a model with loadings constrained across generations to a model with loadings freely estimated showed ΔCFI = 0.012, ΔRMSEA = 0.008, indicating only partial invariance. Therefore, we did not impose equality constraints across generations and instead treated parents and adolescents as distinguishable dyad members, estimating all paths separately for each generation.

### 2.8 Statistical analysis

Primary analysis was performed in IBM AMOS 29. The APIM path model was specified with observed variables. Autoregressive, actor, and partner coefficients were estimated as described in Section 2.4. Covariates (offspring age, parent gender, socioeconomic status, refugee status) were regressed on all endogenous variables (ER at T2 and T3 for both dyad members).

Model fit: Because the basic APIM with three waves can be saturated (zero degrees of freedom) in some specifications, we focused on the significance and magnitude of individual paths. For the mediated models (H5), we examined χ^2^, CFI, RMSEA, and SRMR. Acceptable fit was defined as CFI ≥ 0.95, RMSEA ≤ 0.06, SRMR ≤ 0.08.

Missing data: FIML in AMOS used all available data points. Auxiliary variables (parent education, number of household members) were included to strengthen the missing-at-random assumption.

Power for indirect effects: With *N* = 247 dyads, power exceeded 0.80 for detecting indirect effects of small-to-medium magnitude (*ab* ≥ 0.10) in an APIM framework (Ledermann et al., 2011). Indirect effects (H5) were tested using bias-corrected bootstrap with 5,000 resamples (Preacher and Hayes, 2008).

Software: IBM AMOS 29 (Arbuckle, 2021) with SPSS 29 for data management and preliminary analyses.

### 2.9 Ethical approval and pre-registration

The study protocol was approved by the Institutional Review Board (IRB) of Jadara University, Irbid, Jordan (approval number: 2025/123-27/IRB, date: 15 March 2025). All procedures complied with the Helsinki Declaration (2013 revision). For refugee participants, additional verbal consent in Arabic was obtained, and community liaisons were present to address any concerns about coercion or perceived obligations to participate. Data collection took place from September 2025 to March 2026. The datasets generated and analyzed during the current study are available from the corresponding author upon reasonable request after publication.

## 3 Results

### 3.1 Attrition and missing data

Of the 250 dyads recruited at baseline (T1, September 2025), 247 provided complete data at T1 (three dyads withdrew before any assessment). At T2 (December 2025), 241 dyads were retained (97.6% of the 247). At T3 (March 2026), 236 dyads completed all measures (retention rate = 94.4% from T1). Attrition analysis using independent *t -tests* and *χ^2^* tests revealed no significant differences between completers (*n* = 236) and non-completers (*n* = 11) on any baseline variable: parent trauma (*t* (245) = 0.72, *p* = 0.472), parent ER [*t* (245) = 0.54, *p* = 0.590), offspring ER (*t* (245) = 0.93, *p* = 0.353), offspring age (t (245) = 0.31, *p* = 0.757), parent gender (*χ^2^* = 0.28, *p* = 0.597), or refugee status (*χ^2^* = 0.11, *p* = 0.742).

### 3.2 Descriptive statistics and bivariate correlations

Means, standard deviations, and bivariate correlations for all study variables are presented in Table 1. Parental trauma (T1) showed small to moderate positive correlations with parent ER difficulties at T1 (*r = 0.38, *p < 0.01) and T2 (*r = 0.35, *p < 0.01), and with offspring ER difficulties at T2 (*r = 0.29, *p < 0.01) and T3 (*r = 0.24, *p < 0.01). Offspring ER at T1 was positively correlated with parent ER at T2 (*r = 0.18, *p < 0.05), supporting preliminary evidence of bidirectionality. All correlations were in the expected direction, and no multicollinearity concerns emerged (all *r '*s* < 0.70).

**Table 1:** Means, standard deviations, and bivariate correlations among study variables (*N* = 247 Dyads).

Variable	*M*	SD	1	2	3	4	5	6
1. Parent Trauma (T1)	28.43	12.16	---					
2. Parent ER (T1)	48.27	15.84	0.38**	---				
3. Parent ER (T2)	45.91	16.02	0.35**	0.61**	---			
4. Offspring ER (T1)	52.65	14.93	0.16*	0.21**	0.19*	---		
5. Offspring ER (T2)	51.02	15.66	0.29**	0.25**	0.27**	0.58**	---	
6. Offspring ER (T3)	49.87	16.23	0.24**	0.22**	0.30**	0.54**	0.67**	---

ER, difficulties in emotion regulation (DERS-SF total score); Parent trauma, PCL-5 total score at T1. M, mean; SD, standard deviation.

*p < 0.05; **p < 0.01 (two-tailed).

Regarding covariates, refugee status (Syrian refugee vs. Jordanian citizen) was significantly associated with higher baseline parental trauma [*t (245) = 4.12, *p < 0.001, Cohen’s *d = 0.53], but it was not significantly correlated with any cross-lagged partner effect path (all *r 's < |0.08|, *p > 0.20). When refugee status was entered as a moderator in a separate multi-group APIM (refugee vs. host-community dyads), the critical partner effect from parent trauma T1 to offspring ER T2 did not differ between groups [Δχ^2^ (1) = 1.21, *p = 0.271]. This suggests that the intergenerational transmission mechanism operates consistently across both refugee and host-community dyads, supporting the generalizability of the findings.

### 3.3 Model fit of the longitudinal APIM

The hypothesized three-wave distinguishable dyad APIM (as specified in Section 2.4) was tested using AMOS 29 with maximum likelihood estimation and FIML. The model included autoregressive paths, actor effects (parent trauma T1 → parent ER T2), partner effects (parent trauma T1 → offspring ER T2; offspring ER T1 → parent ER T2), and longitudinal cascades (parent ER T2 → offspring ER T3; offspring ER T2 → parent ER T3). Covariates (offspring age, parent gender, SES, refugee status) were regressed on all endogenous variables.

### 3.4 Hypothesis testing: autoregressive, actor, and partner paths

Table 2. presents unstandardized (*B*), standardized (*β*) coefficients, standard errors, *p* -values, and 95% confidence intervals (for partner and mediation effects) for all hypothesized paths.

**Table 2:** Longitudinal APIM Path Coefficients (*N* = 247 Dyads).

Hypothesis	Path	Type	*B*	*β*	*SE*	*p*	95% CI
H1	Parent ER T1 → Parent ER T2	AR	0.59	0.58	0.06	<0.001	---
H1	Parent ER T2 → Parent ER T3	AR	0.64	0.62	0.07	<0.001	---
H1	Offspring ER T1 → Offspring ER T2	AR	0.57	0.55	0.06	<0.001	---
H1	Offspring ER T2 → Offspring ER T3	AR	0.62	0.60	0.06	<0.001	---
H2	Parent Trauma T1 → Parent ER T2	Actor	0.23	0.19	0.07	0.002	[0.09, 0.37]
H3	Parent Trauma T1 → Offspring ER T2	Partner	0.29	0.23	0.08	<0.001	[0.14, 0.44]
H4	Offspring ER T1 → Parent ER T2	Partner	0.12	0.12	0.06	0.043	[0.01, 0.23]
Explore.	Parent ER T1 → Offspring ER T2	Partner	0.08	0.08	0.06	0.187	[−0.04, 0.20]
Explore.	Parent ER T2 → Offspring ER T3	Partner	0.15	0.15	0.06	0.009	[0.04, 0.26]
Explore.	Offspring ER T2 → Parent ER T3	Partner	0.06	0.06	0.06	0.299	[−0.05, 0.17]

AR, autoregressive path; B, unstandardized coefficient; β, standardized coefficient; SE, standard error; CI, confidence interval (bootstrap percentile, 5,000 resamples). All models controlled for offspring age, parent gender, SES, and refugee status. ER, Difficulties in Emotion Regulation (higher scores = greater difficulties). AR paths for H1 are reported without CIs for brevity; all AR paths were significant at *p* < 0.001.

#### 3.4.1 Autoregressive stability (H1)

As predicted, emotion regulation difficulties showed moderate to strong stability across waves. For parents, ER(T1) → ER(T2) (*β* = 0.58, *p* < 0.001) and ER(T2) → ER(T3) (*β* = 0.62, *p* < 0.001). For offspring, ER(T1) → ER(T2) (*β* = 0.55, *p* < 0.001) and ER(T2) → ER(T3) (*β* = 0.60, *p* < 0.001). All autoregressive coefficients exceeded the hypothesized *β* ≥ 0.40 threshold, supporting H1.

#### 3.4.2 Actor effect of parental trauma (H2)

Parental trauma severity at T1 significantly predicted parents’ own ER difficulties at T2, controlling parent ER at T1 (*β* = 0.19, *p* = 0.002). This actor effect indicates that parents with higher trauma exposure experienced a relative increase in emotion regulation difficulties over the first three months, beyond their baseline level. H2 was supported.

#### 3.4.3 Partner effects—intergenerational transmission (H3)

The critical test of trauma transmission: Parental trauma at T1 significantly predicted offspring ER difficulties at T2 (*β* = 0.23, *p* < 0.001). This partner effect remained significant after controlling for offspring ER at T1, offspring age, gender, SES, and refugee status. The magnitude (*β* = 0.23) represents a small-to-medium effect, consistent with prior longitudinal dyadic studies. Thus, H3 was supported: higher parental unresolved trauma predicted worse offspring emotion regulation three months later.

#### 3.4.4 Bidirectional association—offspring-to-parent (H4)

Offspring ER difficulties at T1 significantly predicted parent ER difficulties at T2 (*β* = 0.12, *p* = 0.043). This effect, although statistically significant, is modest in magnitude and should be interpreted with appropriate caution. Importantly, this effect did not persist to T3 (offspring ER T2 → parent ER T3: *β* = 0.06, *p* = 0.299), indicating that the child-driven effect was limited to a short-term prospective association rather than a stable reciprocal process. This pattern provides only limited evidence of bidirectionality and is insufficient to establish a stable child-driven influence on parental regulation over time. H4 was supported in the short term but not longitudinally.

#### 3.4.5 Additional partner effects (exploratory)

Parent ER at T1 did not significantly predict offspring ER at T2 (*β* = 0.08, *p* = 0.187), suggesting that the direct pathway from parent trauma to offspring ER is stronger than the pathway via parent ER. However, parent ER at T2 significantly predicted offspring ER at T3 (*β* = 0.15, *p* = 0.009), indicating a delayed cascade. Offspring ER at T2 did not significantly predict parent ER at T3 (*β* = 0.06, *p* = 0.299).

### 3.5 Mediation (longitudinal cascade – H5)

We tested whether parent ER at T2 mediated the effect of parent trauma (T1) on offspring ER at T3, and whether offspring ER at T2 mediated the effect of offspring ER (T1) on parent ER at T3, using bias-corrected bootstrap with 5,000 resamples.

#### 3.5.1 Parent-driven cascade

The indirect effect of parent trauma T1 → parent ER T2 → offspring ER T3 was significant [*B* = 0.035, *β* = 0.029, 95% CI (0.010, 0.068), *p* = 0.008], with the direct effect (parent trauma T1 → offspring ER T3) dropping from *β* = 0.19 (*p* = 0.002) to *β* = 0.12 (*p* = 0.057) after including the mediator. The proportion mediated was 19.4%, indicating partial mediation.

#### 3.5.2 Offspring-driven cascade

The indirect effect of offspring ER T1 → offspring ER T2 → parent ER T3 was not significant [*B* = 0.018, *β* = 0.015, 95% CI (−0.007, 0.052), *p* = 0.142], failing to support the child-driven longitudinal cascade. Only the direct path of offspring ER T1 → parent ER T2 (H4) was significant, but this did not propagate to T3.

Thus, H5 was supported only for the parent-to-child direction, not for the reverse direction.

### 3.6 Summary of results

- H1 (autoregressive stability): Supported (strong stability in both parents and offspring).
- H2 (actor effect of parent trauma): Supported (parent trauma predicted worsening of parent’s own ER).
- H3 (partner effect, parent→offspring): Supported (primary evidence of intergenerational transmission).
- H4 (bidirectional partner effect, offspring→parent): Supported (child dysregulation predicted parent dysregulation at T2, but not beyond).
- H5 (longitudinal cascade mediation): Partially supported (parent ER mediated parent trauma → offspring ER at T3; offspring ER did not mediate the reverse).

Figure 1 presents the final path diagram with standardized coefficients. All estimated paths are displayed, with significant paths (*p* < 0.05) shown in black and nonsignificant paths (*p* ≥ 0.05) shown as gray dashed lines with “n.s.” indicated. Autoregressive paths are displayed as solid gray arrows, actor effects as solid black arrows, and partner effects as dashed black arrows. The figure illustrates the mutual dynamics: a moderate parent-to-offspring prospective association (*β* = 0.23) and a smaller but significant offspring-to-parent association (*β* = 0.12) at the first cross-lag.

**Figure 1:**
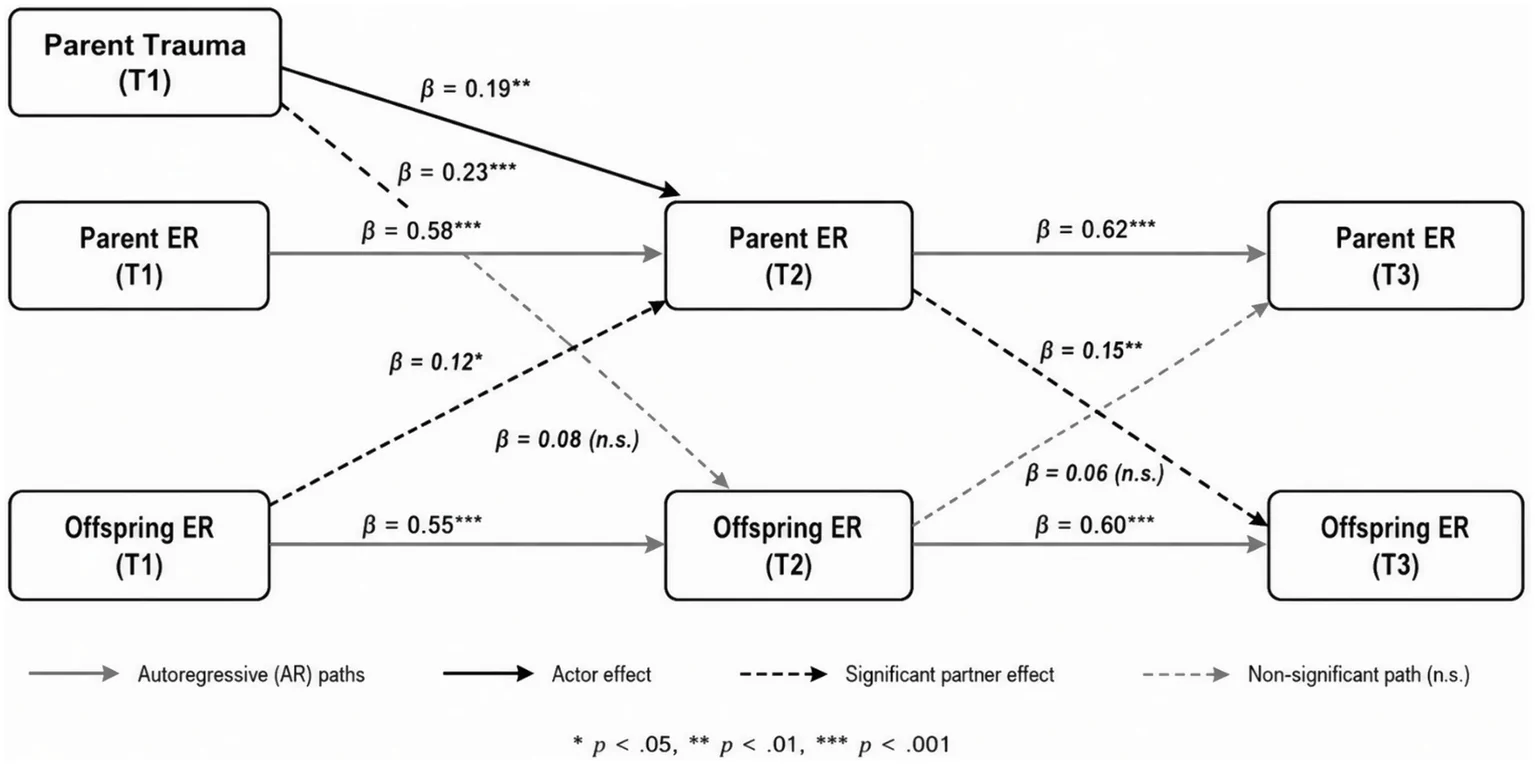
Final cross-lagged path model with standardized coefficients. *Solid black arrows indicate actor effects (*p* < 0.05). Dashed black arrows indicate significant partner effects. Gray dashed arrows indicate nonsignificant paths (*p* ≥ 0.05). Autoregressive paths are shown as solid gray arrows. Within-wave residual covariances between parent ER and offspring ER are included but not shown for visual clarity. Variances for all variables and residuals are available from the corresponding author upon reasonable request. Covariates (offspring age, parent gender, SES, refugee status) were regressed on all endogenous variables but are not shown for visual clarity. *p < 0.05, **p < 0.01, *p* < 0.001. n.s., not significant.

## 4 Discussion

The present study represents a methodological and theoretical pivot in the study of intergenerational trauma. By deploying a three-wave longitudinal Actor-Partner Interdependence Model (APIM) within a culturally diverse Middle Eastern sample, this study moved beyond static, unidirectional models to capture the recursive, dyadic realities of trauma transmission. The findings offer robust empirical support for the conceptualization of trauma transmission not merely as an inherited vulnerability, but as a dynamic, bidirectionally maintained disruption in dyadic emotion regulation (ER).

The present study does not claim to demonstrate intergenerational trauma transmission in the sense of direct transfer of trauma from parent to child. Rather, we provide evidence of prospective associations between parental trauma-related symptoms and adolescent emotion regulation difficulties. These associations are consistent with theoretical models of intergenerational influence, but they do not establish transmission of trauma itself. Expressions such as ‘inherited trauma,’ ‘embodied legacy,’ or ‘structural transmission’ should be understood as theoretical interpretations rather than empirical findings.

### 4.1 The downward cascade: mechanisms of parent-driven transmission

Consistent with our primary hypotheses (H3 and the parent-driven cascade in H5), parental unresolved trauma significantly predicted a worsening of offspring ER (*β* = 0.23, *p* < 0.001) over a six-month period, mediated sequentially by the parent’s own regulatory difficulties (indirect *β* = 0.029, 95% CI [0.010, 0.068]). This downward cascade confirms that the “embodied legacy” of trauma is actively transmitted through daily relational micro-interactions.

An intriguing finding emerged when comparing the direct versus indirect pathways. The direct path from parental trauma (T1) to offspring ER (T2) was significant (*β* = 0.23), whereas the path from parent ER (T1) to offspring ER (T2) was not (*β* = 0.08, *p* = 0.187). This suggests that the intergenerational impact of trauma is not fully captured by the parent’s concurrent level of emotion regulation difficulties. Rather, trauma may exert its influence through additional, unmeasured behavioral channels such as emotional withdrawal, hostile parenting, or reduced availability factors that directly disrupt the child’s regulatory environment without being fully mediated by the parent’s own self-reported dysregulation. This interpretation aligns with observational studies showing that traumatized parents display specific interpersonal behaviors (e.g., frightening or dissociative caregiving) that predict child outcomes above and beyond the parent’s subjective distress (Clark et al., 2021). Future research should incorporate observational measures of parenting behavior to disentangle these distinct mechanisms.

Theoretically, this can be understood through the lens of emotional modeling and disrupted scaffolding (Morris et al., 2024). Parents with high trauma exposure often operate in a state of chronic hyperarousal or dissociation. Consequently, they are less likely to provide the stable, synchronous “external brain” required to help an adolescent down-regulate high-intensity emotion. Over time, the adolescent internalizes this chaotic or withdrawn parental response, failing to consolidate independent, flexible ER strategies.

Additionally, an exploratory cascade revealed that parental ER difficulties at T2 independently predicted offspring ER at T3 (*β* = 0.15, *p* = 0.009), beyond the direct effect of parent trauma at T1. This suggests that ongoing parental dysregulation not merely the historical trauma continues to shape adolescent outcomes, reinforcing the need for concurrent treatment of parental ER. The fact that this effect propagated significantly to the third wave highlights the enduring, structural impact of parental trauma on adolescent neurobiological and psychological development.

### 4.2 Developmental asymmetry: the “perturbation” vs. “structural” effect

The most striking and novel finding of this study relates to the bidirectional dynamics (H4) and the partial failure of the child-driven longitudinal cascade (H5). As hypothesized, offspring ER difficulties at T1 significantly predicted worsening parent ER at T2 (β = 0.12, *p* = 0.043). However, unlike the parent-driven effect, this upward influence did not cascade to T3.

The child-to-parent pathway (*β* = 0.12, *p* = 0.043) provides limited evidence of a short-term association between adolescent dysregulation and subsequent parental ER difficulties. However, several factors warrant caution in interpreting this as robust evidence of bidirectionality. First, the effect size is small and the *p*-value is close to conventional significance thresholds. Second, the lack of persistence to T3 suggests that any child-driven influence is transient rather than structurally enduring. We therefore characterize this finding as preliminary evidence of a possible short-term perturbation effect rather than definitive evidence of stable bidirectional dynamics.

The modest magnitude and temporal limitation of the child-to-parent effect suggest that while adolescents may temporarily influence parental regulation, parents’ regulatory capacities are more stable and less susceptible to child-driven change over longer timeframes. This developmental asymmetry is consistent with the broader literature on parent–child influence, which typically finds that parent-to-child effects are stronger and more enduring than child-to-parent effects (Klahr and Burt, 2014).

### 4.3 Cultural context and the universality of the transmission mechanism

A critical contribution of this study is the integration of both Syrian refugee and Jordanian host-community dyads. While refugee parents predictably reported higher baseline trauma severities, multi-group analyses revealed that the magnitude of the intergenerational transmission path (the partner effect) did not differ based on refugee status.

This finding is of paramount theoretical importance. Notably, the absence of refugee status as a moderator implies that once parental trauma is present, the relational transmission mechanism operates independently of the original trauma context. In practical terms, a traumatized Jordanian parent from the host community and a traumatized Syrian refugee parent produce similar dyadic regulatory disruptions in their offspring. This finding strengthens the case for transdiagnostic, dyadic interventions that focus on the relational process rather than the specific trauma type. It suggests that while the *origins* of the trauma may differ, the psychological mechanism of transmission via emotion regulation is universal.

### 4.4 Educational and counseling implications

The dyadic and longitudinal nature of these findings necessitates a paradigm shift in clinical interventions. Traditional approaches that treat the dysregulated adolescent as the “identified patient” in isolation are fundamentally flawed, as they ignore the bidirectional feedback loops maintaining the pathology.

Given that parental ER mediates the transmission of trauma to the offspring, interventions must target the dyad. Approaches such as Emotionally Focused Family Therapy (EFFT) or parent management training aimed at enhancing parental reflective functioning are strongly indicated. Furthermore, acknowledging the upward effect (child-to-parent), clinicians must validate the immense emotional toll that dysregulated adolescents take on traumatized parents, providing parents with acute distress-tolerance skills to prevent the temporary “perturbation” from escalating into a hostile family climate.

### 4.5 Limitations and future directions

Despite its methodological rigor, the study has limitations.

First, while three waves over six months effectively capture short-term developmental dynamics, they represent a relatively brief snapshot of a lifelong process. Future research should employ longer intervals spanning the transition from early to late adolescence.

Second, the reliance on self-report measures introduces inherent biases. Integrating observational coding of parent–child conflict discussions or physiological synchrony (e.g., heart rate variability) would provide a multi-method validation of the APIM paths.

Third, while we controlled for several covariates, future models could explore whether genetic or epigenetic vulnerabilities moderate the strength of the transmission paths.

Fourth, while we tested refugee status as a moderator, the sample size (*n* = 247) provided limited power to detect small interaction effects in a multi-group framework; thus, the null finding of no moderation should be interpreted cautiously. Future studies with larger refugee subsamples are needed to confirm the invariance of transmission paths.

Fifth, the APIM was estimated using observed variables rather than latent constructs, which may have attenuated path coefficients due to measurement error; future research should consider latent variable approaches with multiple indicators per wave.

Sixth, the study did not assess several important confounders that may influence both parental trauma-related symptoms and emotion regulation difficulties in both generations. Parental depression, anxiety, current life stress, family conflict, parenting behaviors, and emotional availability are plausible common causes of ER difficulties in parents and adolescents (Clark et al., 2021; Morris et al., 2024). Without measuring these constructs, we cannot rule out the possibility that observed associations are partially or fully explained by these unmeasured third variables. For example, a parent with depression may report both higher trauma-related symptoms and greater ER difficulties and may also engage in parenting behaviors that undermine adolescent ER development, creating a spurious association between parental symptoms and offspring outcomes. Similarly, family conflict may independently predict ER difficulties in both generations, accounting for the observed dyadic associations. While we controlled several demographic factors (offspring age, parent gender, SES, refugee status) and included autoregressive paths that account for baseline levels, residual confounding remains a concern. We therefore caution against interpreting parental ER difficulties as a specific mediating mechanism; rather, our findings should be viewed as demonstrating prospective associations that may be explained by multiple, potentially unmeasured, pathways. Future research should incorporate comprehensive assessments of parental psychopathology, family environment, and observational measures of parenting behavior to disentangle these mechanisms.

Seventh, a significant limitation of the study design is that parental trauma-related symptoms were assessed only at baseline, whereas ER difficulties were measured longitudinally. While the three-wave design allows us to examine prospective associations between T1 parental symptoms and subsequent ER outcomes, it does not allow us to establish the temporal ordering between parental symptoms and ER difficulties fully. Changes in parental symptoms over the six-month period may have preceded or followed changes in parental and offspring ER, but our design cannot test these alternative temporal sequences. Future research should assess trauma-related symptoms at multiple time points to examine bidirectional temporal dynamics between parental symptoms and ER difficulties (Little, 2022).

Finally, while all instruments used in this study have been validated in Arabic-speaking populations generally (Ibrahim et al., 2018; El-Ghossain et al., 2020), they have not been specifically validated in the Syrian refugee or Jordanian host-community populations included in this sample. Cultural differences in the expression of trauma-related symptoms and emotion regulation difficulties may affect the validity of these measures in specific populations. Future research should prioritize the development and validation of culturally adapted instruments for refugee and Middle Eastern populations to ensure accurate assessment across diverse cultural contexts.

## 5 Conclusion

Moving beyond cross-sectional and unidirectional constraints, this study provides robust evidence that intergenerational trauma is a dynamically co-constructed legacy. Parents transmit the shadows of their past through their current regulatory capacities, structurally shaping their children’s emotional futures. Breaking the cycle of inherited trauma which we quantified as a moderate partner effect of *β* = 0.23 from parent to offspring requires healing not just the individual, but the space between them. The bidirectional yet asymmetric dynamics identified here (offspring-driven perturbation at *β* = 0.12) further underscore that adolescents are active agents in family emotional systems, even if their structural impact is developmentally bounded. However, these findings should be interpreted as demonstrating prospective associations rather than causal transmission of trauma itself, and multiple unmeasured confounders may account for some or all of the observed associations.

## Data Availability

The raw data supporting the conclusions of this article will be made available by the authors, without undue reservation, after publication and subject to ethical approval and the protection of participant confidentiality.

## References

[ref1] Ackerman R. A. Ledermann T. Kenny D. A. (2017). Power analysis for the actor-partner interdependence model. J. Fam. Psychol. 31, 111–116. doi: 10.1037/fam0000224, 27668933

[ref2] American Psychiatric Association (2022). Diagnostic and Statistical Manual of Mental Disorders. 5th ed., text rev. Edn Washington, DC, USA: American Psychiatric Association.

[ref3] Arbuckle J. L. (2021). IBM SPSS Amos 29 User's Guide. Chicago, IL, USA: IBM Corporation.

[ref4] Blevins C. A. Weathers F. W. Davis M. T. Witte T. K. Domino J. L. (2015). The posttraumatic stress disorder checklist for DSM-5 (PCL-5): development and initial psychometric evaluation. J. Trauma. Stress. 28, 489–498. doi: 10.1002/jts.22059, 26606250

[ref5] Bowers M. E. Yehuda R. (2016). Intergenerational transmission of stress in humans. Neuropsychopharmacology 41, 232–244. doi: 10.1038/npp.2015.247, 26279078 PMC4677138

[ref6] Chen F. F. (2007). Sensitivity of goodness of fit indexes to lack of measurement invariance. Struct. Equ. Model. 14, 464–504. doi: 10.1080/10705510701301834

[ref7] Clark E. L. M. Jiao Y. Sandoval K. Biringen Z. (2021). Neurobiological implications of parent-child emotional availability: a review. Brain Sci. 11:1016. doi: 10.3390/brainsci11081016, 34439635 PMC8391119

[ref8] Cook W. L. Kenny D. A. (2005). The actor–partner interdependence model: a model of bidirectional effects in developmental studies. Int. J. Behav. Dev. 29, 101–109. doi: 10.1080/01650250444000405

[ref9] Dalgaard N. T. Todd B. K. Daniel S. I. F. Montgomery E. (2020). The transmission of trauma in refugee families: a systematic review and meta-analysis. Eur. J. Psychotraumatol. 11:1735164. doi: 10.1080/20008198.2020.1735164

[ref10] Dashorst P. Mooren T. M. Kleber R. J. de Jong P. J. Huntjens R. J. C. (2019). Intergenerational consequences of the holocaust on offspring mental health: a systematic review of associated factors and mechanisms. Eur. J. Psychotraumatol. 10:1654065. doi: 10.1080/20008198.2019.1654065, 31497262 PMC6720013

[ref11] El-Ghossain M. Al-Sharif M. Al-Masri A. (2020). Arabic validation of the difficulties in emotion regulation scale – short form. Arab J. Psychiatry 31, 112–120.

[ref12] Hallion L. S. Steinman S. A. Tolin D. F. Diefenbach G. J. (2018). Psychometric properties of the difficulties in emotion regulation scale (DERS) and its short forms in adults with emotional disorders. Front. Psychol. 9:539. doi: 10.3389/fpsyg.2018.00539, 29725312 PMC5917244

[ref13] Hamaker E. L. Kuiper R. M. Grasman R. P. P. P. (2015). A critique of the cross-lagged panel model. Psychol. Methods 20, 102–116. doi: 10.1037/a0038889, 25822208

[ref14] Hardt J. Rutter M. (2004). Validity of adult retrospective reports of adverse childhood experiences: review of the evidence. J. Child Psychol. Psychiatry 45, 260–273. doi: 10.1111/j.1469-7610.2004.00218.x, 14982240

[ref15] Henrich J. Heine S. J. Norenzayan A. (2010). The weirdest people in the world? Behav. Brain Sci. 33, 61–83. doi: 10.1017/S0140525X0999152X, 20550733

[ref16] Ibrahim H. Ertl V. Catani C. Ismail A. A. Neuner F. (2018). The validity of posttraumatic stress disorder checklist for DSM-5 (PCL-5) as screening instrument with Kurdish and Arab displaced populations living in the Kurdistan region of Iraq. BMC Psychiatry 18:259. doi: 10.1186/s12888-018-1839-z, 30115040 PMC6097219

[ref17] Isobel S. Goodyear M. Furness T. Foster K. (2019). Preventing intergenerational trauma transmission: a critical interpretive synthesis. J. Clin. Nurs. 28, 1100–1113. doi: 10.1111/jocn.14735, 30556334

[ref18] Kenny D. A. Kashy D. A. Cook W. L. (2006). Dyadic Data Analysis. New York, NY, USA: Guilford Press.

[ref19] Kenny D. A. Ledermann T. (2010). Detecting, measuring, and testing dyadic patterns in the actor-partner interdependence model. J. Fam. Psychol. 24, 359–366. doi: 10.1037/a0019651, 20545409

[ref20] Klahr A. M. Burt S. A. (2014). Elucidating the etiology of direction of effects in parent-child interactions. Psychol. Bull. 140, 1–24. doi: 10.1037/a0036122, 24016230

[ref21] Ledermann T. Macho S. Kenny D. A. (2011). Assessing mediation in dyadic data using the actor-partner interdependence model. Struct. Equ. Model. 18, 595–612. doi: 10.1080/10705511.2011.607099

[ref22] Little T. D. (2022). Longitudinal Structural Equation Modeling. 2nd Edn New York, NY, USA: Guilford Press.

[ref23] Lobo F. M. Lunkenheimer E. (2020). Understanding the parent-child coregulation patterns shaping child self-regulation. Dev. Psychol. 56, 1121–1134. doi: 10.1037/dev0000926, 32309982 PMC7556995

[ref24] Morris A. S. Haines N. DePasquale C. (2024). “The family as a context for emotion regulation development,” in Handbook of Emotion Regulation, eds. Gross J. J. Ford B. Q.. 3rd ed (New York, NY, USA: Guilford Press), 234–251.

[ref25] Preacher K. J. Hayes A. F. (2008). Asymptotic and resampling strategies for assessing and comparing indirect effects in multiple mediator models. Behav. Res. Methods 40, 879–891. doi: 10.3758/BRM.40.3.879, 18697684

[ref26] Putnick D. L. Bornstein M. H. (2016). Measurement invariance conventions and reporting: the state of the art and future directions for psychological research. Dev. Rev. 41, 71–90. doi: 10.1016/j.dr.2016.06.004, 27942093 PMC5145197

[ref27] Reitz E. Deković M. Meijer A. M. (2006). Relations between parenting and externalizing and internalizing problem behaviour in early adolescence: child behaviour as moderator and predictor. J. Adolesc. 29, 419–436. doi: 10.1016/j.adolescence.2005.08.003, 16168474

[ref28] Yehuda R. Daskalakis N. P. Bierer L. M. Bader H. N. Klengel T. Holsboer F. . (2016). Holocaust exposure induced intergenerational effects on FKBP5 methylation. Biol. Psychiatry 80, 372–380. doi: 10.1016/j.biopsych.2015.08.005, 26410355

[ref29] Yehuda R. Lehrner A. (2018). Intergenerational transmission of trauma effects: putative role of epigenetic mechanisms. World Psychiatry 17, 243–257. doi: 10.1002/wps.20568, 30192087 PMC6127768

